# Migration of rigid gas permeable contact lens into the upper eyelid after trauma: a case report

**DOI:** 10.1186/s12886-016-0249-6

**Published:** 2016-06-01

**Authors:** Hyera Kang, Yasuhiro Takahashi, Hirohiko Kakizaki

**Affiliations:** Department of Oculoplastic, Orbital & Lacrimal Surgery, Aichi Medical University Hospital, 1-1 Yazako-Karimata, Nagakute, Aichi 480-1195 Japan; Department of Ophthalmology, University of Seonam College of Medicine, Presbyterian Medical Center, Jeonju, Korea

**Keywords:** Rigid gas permeable contact lens, Migration, Trauma, Subconjunctival space, fibrous capsule, Granulation tissue

## Abstract

**Background:**

Migration of a rigid gas permeable (RGP) contact lens after trauma is rare, and its clinical characteristics have not been fully discussed.

**Case presentation:**

A 36-year-old female showed mild swelling in the right upper eyelid. She lost her RGP contact lens seven months prior to her first visit, from trauma by her child’s kick to the right eye. At the first examination, we felt a firm lump inferior to the right brow. Eversion of the upper eyelid also revealed a firm subconjunctival mass superior to the upper tarsus. After incising the conjunctiva, the RGP contact lens was found without a fibrous capsule and granulation tissue in the subconjunctival space. Three years after removal of the lens, the patient did not show any complications, including ptosis.

**Conclusion:**

The RGP contact lens in the present case migrated into the subconjunctival space superior to the upper tarsus without a fibrous capsule and granulation tissue. These findings are similar to those in previously reported traumatic cases but are different from those in some spontaneous migration cases. This difference may be caused by differences in the migration mechanisms.

## Background

Contact lens migration was first reported in 1963 [1], followed by approximately 50 reported cases [2]. The majority of cases exhibited spontaneous migration [2], while a traumatic migration was rare and only three traumatic cases had been reported [3–5]. The site of migration was variable, including the upper fornix, intra-tarsus, preseptal space, and intra-orbit [2]. Most patients remained asymptomatic for a certain period, but later occasionally complained of chronic irritation, mucous discharge, eyelid swelling, and mechanical ptosis [2, 6].

Here, the following case presents a rare case of a rigid gas permeable (RGP) contact lens migrating to the upper eyelid after trauma.

## Case presentation

Institutional Review Board approval from the Ethics Committee at Aichi Medical University was obtained (No. 15–033), and the tenets of the Declaration of Helsinki were followed. The patient gave informed consent prior to inclusion in the study. Written informed consent was obtained from the patient for publication of this case report and any accompanying images.

A 36-year-old female complained of right upper eyelid swelling. She lost her RGP contact lens 7 months prior to her first visit from trauma by her child’s kick to the right eye. She did not have a prior history of any ophthalmic disease.

**Fig. 1:**
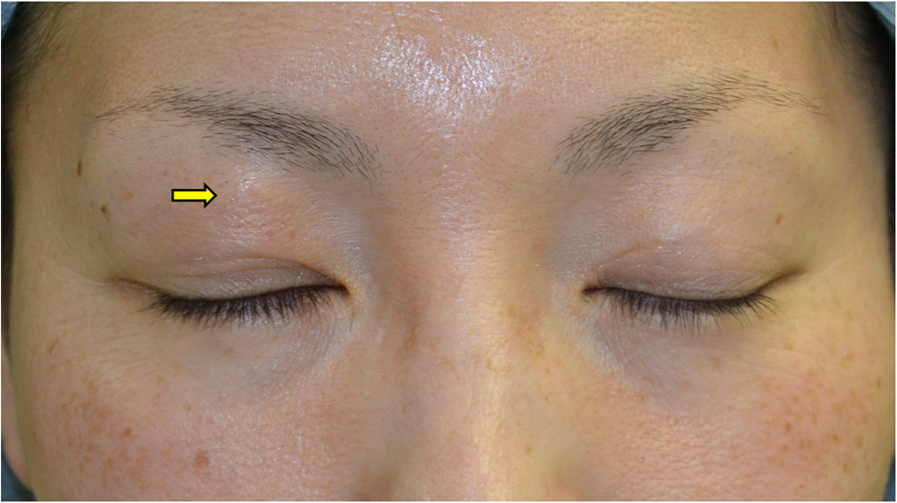
A photograph of the patient’s face. A mass is shown just inferior to the right brow (arrow)

**Fig. 2:**
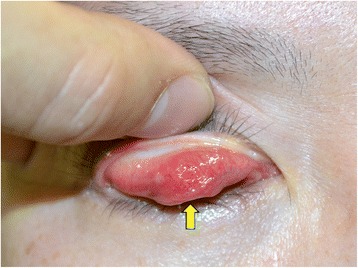
A photograph of the patient’s face after eversion of the upper eyelid. A mass is located superior to the upper tarsus (arrow)

At the first examination, her best-corrected visual acuity was 1.5 OU. A firm lump was palpated inferior to the right brow (Fig. 1). Eversion of the upper eyelid also revealed a firm subconjunctival mass superior to the upper tarsus (Fig. 2). The patient did not show ptosis at that time. Axial computed tomographic images showed a non-cystic mass in the right upper eyelid.

**Fig. 3:**
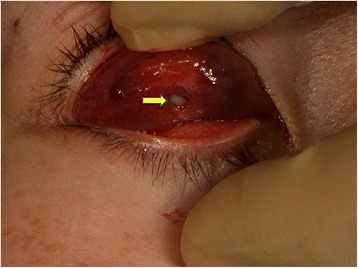
Intraoperative findings after a conjunctival incision. A rigid gas permeable contact lens (arrow) was found in the subconjunctival space, without a fibrous capsule or granulation tissue

**Fig. 4:**
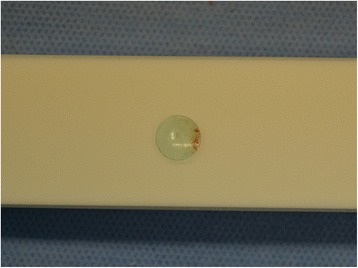
A photograph of the rigid gas permeable contact lens. The extracted contact lens is shown

The mass was removed via a conjunctival incision under local anesthesia. A RGP contact lens was found in the subconjunctival space with a mucopurulent pus discharge (Figs. 3 and 4). There was no fibrous capsule or granulation tissue around the RGP contact lens. No microorganisms were isolated from the discharge. Three years after the removal, the patient showed no complications, including decreased vision and ptosis.

## Conclusions

**Fig. 5:**
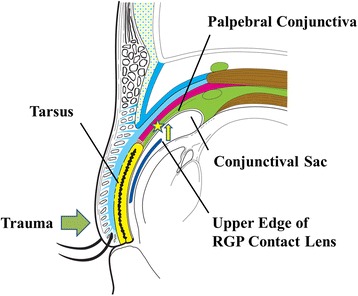
Schema of the sagittal section of the orbit. The upper conjunctival sac curves superoposteriorly along the sclera. During eyelid closure, the upper edge of a rigid gas permeable (RGP) contact lens is located below the palpebral conjunctiva, superior to the upper tarsus (asterisk). When the lens is pushed upwardly (yellow arrow) by trauma, the upper edge penetrates the palpebral conjunctiva, superior to the upper tarsus

This case report describes a rare case of a RGP contact lens migration as a result of trauma. The RGP contact lens in the present report migrated into the subconjunctival space superior to the upper tarsus. The position of a migrated lens may be associated with physiological and anatomical factors. The eyes close by blink reflexes [7], and a RGP contact lens on the cornea may be slightly shifted upwards by Bell’s phenomenon immediately before a blunt ocular trauma. At the time, the upper edge of a RGP contact lens was located below the palpebral conjunctiva, as the upper conjunctival sac curves superoposteriorly along the sclera (Fig. 5) [8]. The lens was therefore pushed upward by the force of trauma, penetrating the palpebral conjunctiva at the point.

Similar case reports with migration caused by trauma have been reported, with all sites involving the subconjunctival space above the upper tarsus [3–5]. However, previous reports of spontaneously migrating contact lenses described a variety of migration locations, such as the intra-tarsus, preseptal space, and intra-orbit [2]. A contact lens simply penetrates the conjunctiva in traumatic cases; in contrast, chronic abrasion of periocular tissue by a contact lens induces local necrosis or abscess in spontaneous cases [2, 9]. Periocular tissue disruption may cause migration into hard tissues or deeper spaces in spontaneous cases.

In the present case, no fibrous capsule or granulation tissue was seen around the migrated RGP contact lens. This finding is similar to previous reports of traumatic cases [3–5]. However, capsule and/or granulation tissue have been found in most spontaneous migration cases [2]. Traumatic cases show less tissue disruption during the migration process than spontaneous cases. This difference may be associated with variations in the severity of the inflammatory responses in the periocular tissue around the migrated contact lenses.

In conclusion, this case report describes a rare case of RGP contact lens migration resulting from trauma to the upper eyelid fornix. Clinical findings may differ between traumatic and spontaneous cases because of differences in the migration mechanisms.

## Abbreviations

RGP, rigid gas permeable
